# The Elasticity Coefficients Measurement of Human Dentin Based on RUS

**DOI:** 10.1155/2017/7852971

**Published:** 2017-04-30

**Authors:** Fan Fan, Dandan Feng, Rui Wang, Qiang Zhang, Haijun Niu

**Affiliations:** Key Laboratory of Ministry of Education for Biomechanics and Mechanobiology, School of Biological Science and Medical Engineering, Beihang University, Beijing, China

## Abstract

This paper proposed to take advantages of resonant ultrasound spectroscopy (RUS) to measure the mechanical properties of human dentin specimen. The resonant spectroscopy of the dentin specimen was obtained between the frequency bands 155 and 575 kHz, and resonant frequencies were extracted by linear predictive filter and then by Levenberg-Marquardt method. By inverse problem approach, 13 experimental resonant frequencies progressively matched to the first 30 orders of theoretical resonant frequencies calculated by Lagrangian variational method. The full second-order elastic tensor of dentin specimen was adjusted. The whole set of human dentin engineering moduli, including Young's moduli (*E*_11_ = 22.641 GPa, *E*_33_ = 13.637 GPa), shear moduli (*G*_12_ = 10.608 GPa, *G*_23_ = 7.742 Gpa), and Poisson's ratios (*ν*_12_ = 0.067, *ν*_31_ = 0.378), were finally calculated. This study demonstrates that RUS can be successfully adapted to measure the mechanical properties of low quality factor biomaterials.

## 1. Introduction

As the most abundant mineralized tissue in human teeth, dentin is composed of about 50% inorganic components (basically calcium hydroxyapatite), 30% organic components (mainly type I collagen fibers), and 20% water [1, 2]. Compared with enamel, dentin has less inorganic components and thus is softer and more elastic. These characteristics ensure dentin being indispensable to cushion chewing force and protect internal pulp [3].

The researches about dentin mechanical properties began from 19th century and never stopped since then [3]. To date, the main methods of dentin mechanical properties measurement mainly included tensile and compression test [2, 4], acoustics method (pulse echo method, bulk wave method, elasticity imaging, etc.) [4–8], and macroscopic indentation and nanoindentation method [9–11]. These methods helped not only promote the understanding of macromechanical properties of dentin and micromechanical properties of dentin tubule, but offer important significance for the design, development, and evaluation of clinical dental restorative materials as well. Nevertheless, these methods mentioned above have their own limitations. For example, the macrotest methods required a relative larger size of the specimen, which was difficult to obtain from human dentin. Besides, although the nanoindentation method could be used to measure the elastic moduli in different directions of small-sized specimen, it mainly reflected that the mechanical properties deeply rely on the scope of indentation, which led to the differences from the macroscopic mechanical properties.

Since the 1990s, resonant ultrasound spectroscopy (RUS) has been developed as an accurate and efficient method to characterize the material properties [12–15]. The basic mechanism of RUS is to obtain a series of mechanical resonant frequencies by generating free vibrations with ultrasound excitations, then predict theoretical model frequencies with Lagrangian variational method, and finally get the material elastic properties by comparing the measured frequencies with the predicted ones (inverse problem approach). RUS has been regarded as the gold standard for measuring the elastic moduli of high *Q* (quality factor) solid materials. And it beats other methods by the following three advantages: ① The full elastic tensor could be assessed from a single sample in a single nondestructive experiment over other elasticity measurement methods; ② RUS was well-adapted to small-sized samples (a few millimeters or less); ③ The measurement results were more repeatable and accurate [12, 14, 16]. In recent years, Pascal's group were dedicated to measuring the elastic coefficients of cortical bone by RUS, which made it possible for breaking the limitations when measuring low-*Q* materials by RUS [12, 13, 17].

In this paper, the method of human dentin elasticity extraction based on RUS was studied. Firstly, the resonant spectroscopy of dentin specimen was obtained by ultrasound experiment. Then, the resonant frequencies were extracted through signal processing of linear predictive filter and then by nonlinear least squares method (Levenberg-Marquardt method). Combined with the theoretical resonant frequencies calculated by Lagrangian variational method, inverse problem approach was introduced to obtain the complete second-order elastic tensor of dentin samples. The engineering moduli, including Young's moduli, shear moduli, and Poisson's ratios, were finally calculated.

## 2. Materials and Methods

### 2.1. Specimen Preparations

The tooth used in this paper was complete, fresh, and caries-free. It was a left upper third molar from a 25-year-old male, which was collected from Beihang Hospital. The donor provided consent to donate his tooth for this study. The entire experimental procedures were approved by the Institutional Animal Care and Use Committee of Beihang University and performed under the guidelines of the National Institutes of Health.

Before experimentation began, the specimen was stored in saline solution at room temperature (22°C). After cleaning the entire tooth surface by removing calculus and granulation, the dentin part was cut into a rectangular parallelepiped by a low speed diamond cutting machine (SYJ-150, Shenyang Kejing Auto-Instrument Co., Ltd., Shenyang, China) with 0.01 mm positioning accuracy and 25–300 rad/min rotational speed and a whole sintered diamond saw blade (0.3 mm thick). The six faces of the specimen were polished with P500, P800, and P1000 abrasive paper progressively. With repeated protractor measurements of each corner of the specimen and polish, a standard rectangular parallelepiped human dentin specimen was obtained (accuracy: 90° ± 0.5°) [12]. The mass of the specimen was 111 mg, and the dimensions were 5.696 mm × 3.620 mm × 2.704 mm.

### 2.2. Theoretical Resonant Frequencies Calculation

The resonant frequencies of solid material are related to many factors such as density, dimensions, elastic tensor, and boundary conditions. Moreover, the relationship among these factors is nonlinear and no analytical solutions exist either. To figure out the approximate numerical solutions, Lagrange variational method was imported here. As (1) shows, the resonant angular frequencies *ω* could be calculated by searching for the stationary points of the Lagrangian *L* under free-surface boundary condition [14, 18–21].

(1) $$\begin{array}{c} L=\int \left(\;E_{k}-E_{p}\;\right)dV, \end{array}$$

where *E*_*k*_ and *E*_*p*_ are kinetic energy and potential energy, respectively, given by

(2) Ek=12∑iρω2ui2,Ep=12∑i,j,k,lcijkl∂ui∂xj∂uk∂xl.

In (2), *ρ* and *V* are the specimen's density and volume, respectively, *u*_*i*_ is the component of the displacement field in Cartesian coordinates, and *c*_*ijkl*_ are the stiffness constants of solid material.

Kinney et al. found the elastic constants *C*_*ij*_ of hydrated dentin exhibited as transverse isotropy, with five independent constants: *C*_11_, *C*_12_, *C*_13_, *C*_33_, and *C*_44_, as (3) shows [1, 22]:

(3) $$\begin{array}{c} C_{ij}=\left(\begin{array}{cccccc} C_{11} & C_{12} & C_{13} & 0 & 0 & 0\\ C_{12} & C_{11} & C_{13} & 0 & 0 & 0\\ C_{13} & C_{13} & C_{33} & 0 & 0 & 0\\ 0 & 0 & 0 & C_{44} & 0 & 0\\ 0 & 0 & 0 & 0 & C_{44} & 0\\ 0 & 0 & 0 & 0 & 0 & \frac{1}{2}\left(C_{11}-C_{12}\right) \end{array}\right). \end{array}$$

To find the stationary point of the Lagrangian *L*, (4) was calculated:

(4) $$\begin{array}{c} \delta L=0. \end{array}$$

To solve the numerical solutions of (4), by expanding the displacement field *u*_*i*_ to a set of polynomial functions, Rayleigh-Ritz method was introduced:

(5) $$\begin{array}{c} u_{i}=\underset{\lambda }{\sum }a_{i\lambda }\phi_{\lambda }, \end{array}$$

where the choice of *ϕ*_*λ*_ is rather arbitrary.

Historically, Visscher et al. found that there was none simpler than powers of the Cartesian coordinates when expanding the displacement field [18], so a set of power exponent functions were chosen for simplifying computing:

(6) $$\begin{array}{c} \phi_{\lambda }=x^{l}y^{m}z^{n}, \end{array}$$

where *l* + *m* + *n* ≤ *N*.

When *N* → +*∞*, the solutions of (4) are the exact solutions. Considering a good compromise between computational accuracy and computing time, *N* was chosen as 12.

Based on (5) and (6), (4) was transferred to the generalized eigenvalue problem:

(7) $$\begin{array}{c} \omega^{2}Ea=\Gamma a, \end{array}$$

where *E* and Γ are expressed as follows:

(8) Eλiλ′i′=δii′ρ∫Vxl+l′ym+m′zn+n′dV,Γλiλ′i′=∑j,j′Ciji′j′∫V∂xlymzn∂xj∂xl′ym′zn′∂xj′dV.

In the end, the theoretical values of solid resonant frequency could be calculated by solving (7).

Before the resonance experiment, a set of initial elastic tensors combined with the density and dentin specimen dimensions were needed to be set; then the theoretical resonant frequencies could be calculated later. The initial elastic tensor set plays a decisive role in iterative efficiency and accuracy, so it is indispensable to find an exact-value-closest set as shown in matrix (9) [18, 22]. In this paper, the first 30 frequencies' range was chosen as the experimental frequency sweep range reference.

(9) $$\begin{array}{c} \left(\begin{array}{cccccc} 42.6 & 25.4 & 19.7 & 0 & 0 & 0\\ 25.4 & 42.6 & 19.7 & 0 & 0 & 0\\ 19.7 & 19.7 & 34.6 & 0 & 0 & 0\\ 0 & 0 & 0 & 9.4 & 0 & 0\\ 0 & 0 & 0 & 0 & 9.4 & 0\\ 0 & 0 & 0 & 0 & 0 & 8.6 \end{array}\right). \end{array}$$

### 2.3. RUS Experiment

**Figure 1:**
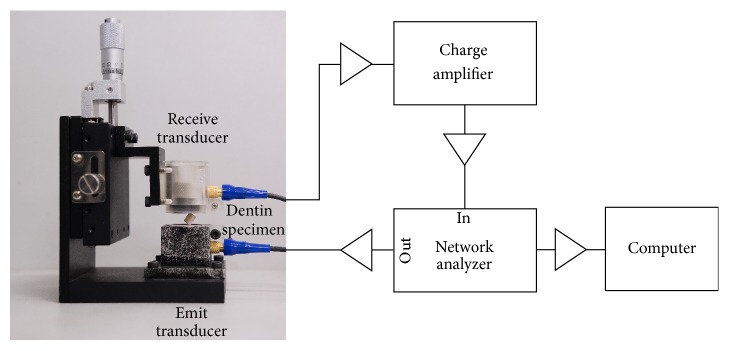
RUS experiment platform: photograph of the dentin specimen mounted between emitting and receiving transducers and block diagram of other hardware.

The RUS experiment platform is shown in Figure 1. The platform was made just to fit the free-surface boundary condition. The dentin specimen was mounted on opposing corners between two shear wave contact transducers (V154RM, Panametrics Inc., Waltham, US) in the RUS system. A network analyzer (Bode 100, Omicron electronics GmbH, Klaus, Austria) was used to output a swept-frequency signal between 155 kHz and 575 kHz (frequency resolution: 30 Hz) as the excitation of the transmit transducer. The frequency response of the specimen was received by the other transducer, amplified by a broadband charge amplifier (HQA-15M-10T, Femto Messtechnik GmbH, Berlin, Germany), sent back to the network analyzer, and recorded [12].

### 2.4. Experimental Resonant Frequencies Extraction

The frequency response FR acquired by the network analyzer was modeled as a sum of *M* Lorentzian lineshapes [12]:

(10) $$\begin{array}{c} FR\left(\;f\;\right)=\overset{M}{\underset{k=1}{\sum }}\frac{a_{k}}{\left({f_{k}}^{2}-f^{2}\right)+i\left(f_{k}f/Q_{k}\right)}, \end{array}$$

where *a*_*k*_ are the complex amplitudes, *f*_*k*_ are the resonant frequencies, and *Q*_*k*_ are the resonant quality factors.

When RUS was used on low damping (high *Q*) materials, the resonant frequencies *f*_*k*_, the sharp peak, could be easily recognized from the resonant spectrum. For those low *Q* materials such as dentin, peaks may be broad and overlap each other, which made it difficult to directly extract resonant frequencies from spectrum. Therefore linear predictive filter, an accurate signal processing method, introduced by Kumaresan, Tufts, and Lebedev et al. was selected here to distinguish the resonant frequencies, which was proved to be a perfect solution [23–27].

The frequency response FR was converted to *y*(*n*) in time domain by *N*-point inverse Fourier transform (*N* is the length of the FR data), and then matrix *A* was created based on

(11) $$\begin{array}{c} A=\left(\begin{array}{cccc} y\left(L\right) & y\left(L-1\right) & \cdots & y\left(1\right)\\ y\left(L+1\right) & y\left(L\right) & \cdots & y\left(2\right)\\ \vdots & \vdots & \ddots & \vdots \\ y\left(N-1\right) & y\left(N-2\right) & \cdots & y\left(N-L\right) \end{array}\right). \end{array}$$

According to the linear prediction method, the first *L* points of *y*(*n*) contained enough information to predict values of the others. So the linear predictive filter equation could be set as

(12) $$\begin{array}{c} Ag=b, \end{array}$$

where *b* has the definition:

(13) $$\begin{array}{c} b=\left(\begin{array}{c} \begin{array}{c} y\left(L+1\right)\\ y\left(L+2\right) \end{array}\\ \vdots \\ y\left(N\right) \end{array}\right) \end{array}$$

and *g* is a column vector with *L* components:

(14) $$\begin{array}{c} g=\left(\begin{array}{c} \begin{array}{c} g_{1}\\ g_{2} \end{array}\\ \vdots \\ g_{L} \end{array}\right). \end{array}$$

Then the transfer function of this linear predictive filter could be written as

(15) $$\begin{array}{c} H\left(\;z\;\right)=1+\overset{L}{\underset{k=1}{\sum }}g_{k}z^{-k}. \end{array}$$

The predicted values of the resonant frequency *f*_*k*_ and the quality factor *Q*_*k*_ could be obtained by finding the zeros outside the unit circle in *Z* domain. Substituting the predicted *f*_*k*_ and *Q*_*k*_ into (10), complex resonant amplitude *a*_*k*_ and also the predicted frequency spectrum FR*lp* could be calculated. One of the advantages of this method was that there was no need to know the exact numbers of resonant peaks in FR, but some differences existed between FR*lp* and FR. To figure out these differences, a Levenberg-Marquardt method was taken to optimize the parameters, and we considered the *f*_*k*_'s which minimized (16), the true resonant frequencies [28].

(16) $$\begin{array}{c} F\left(\;f\;\right)=\sum {\left(\;\frac{FRlp\left(f\right)-FR\left(f\right)}{FR\left(f\right)}\;\right)}^{2}. \end{array}$$

During a single experiment, some resonant mode might not be excited, so 7 measurements on the dentin specimen, by remounting it on opposing corners each time, were conducted for a good reproducibility. Each of the regarded experimental resonant frequencies *f*^exp^ was present in at least two frequency responses in the 7 measurements.

### 2.5. Elastic Tensor Calculation

Levenberg-Marquardt nonlinear optimization inverse problem approach was selected for the purpose of calculating the elastic tensor [14, 28, 29]. Here the cost function was introduced as a criterion for the iteration, and the value of the cost function was calculated, as shown in

(17) $$\begin{array}{c} F\left(\;C\;\right)=\overset{N}{\underset{i=1}{\sum }}{w_{i}\left(f_{i}^{exp}-f_{i}^{cal}\left(C\right)\right)}^{2}, \end{array}$$

where *C* is an independent component of the elastic tensor, *N* is the number of the resonant frequency, *f*_*i*_^cal^ is the *i*th order calculated resonance frequency, *f*_*i*_^exp^ is the *i*th order experimental resonance frequency, and *w*_*i*_ is weighting factor, expressed as follows:

(18) $$\begin{array}{c} w_{i}=\left\{\begin{array}{cc} 0 & f_{i}^{cal}\text{ does not match }f_{i}^{exp}\\ \frac{1}{{\left(f_{i}^{exp}\right)}^{2}} & f_{i}^{cal}\text{ matches}\;\;f_{i}^{exp}. \end{array}\right. \end{array}$$

The iterative process was completed when the experimental and theoretical frequencies matched perfectly. In other words, cost function reached the global minimum and also became convergent.

### 2.6. Engineering Moduli Calculation

The 6 × 6 stiffness matrix was constructed and numerically inverted to obtain the compliance matrix *C*_*ij*_^−1^, from which engineering moduli could be calculated.

(19) $$\begin{array}{c} C_{ij}^{-1}=\left(\begin{array}{cccccc} \frac{1}{E_{11}} & \frac{-\nu_{12}}{E_{11}} & \frac{-\nu_{31}}{E_{33}} & 0 & 0 & 0\\ \frac{-\nu_{12}}{E_{11}} & \frac{1}{E_{11}} & \frac{-\nu_{31}}{E_{33}} & 0 & 0 & 0\\ \frac{-\nu_{31}}{E_{33}} & \frac{-\nu_{31}}{E_{33}} & \frac{1}{E_{33}} & 0 & 0 & 0\\ 0 & 0 & 0 & \frac{1}{G_{23}} & 0 & 0\\ 0 & 0 & 0 & 0 & \frac{1}{G_{23}} & 0\\ 0 & 0 & 0 & 0 & 0 & \frac{1}{G_{12}} \end{array}\right), \end{array}$$

where *E*_*ii*_'s are Young's moduli (GPa), *G*_*ij*_'s are shear moduli (GPa), and *ν*_*ij*_'s are Poisson's ratios.

## 3. Result and Discussion

### 3.1. Results of Experimental Resonant Frequencies Extractions

In accordance with the method of 2.4, in each of 7 measurements, two or more resonant frequencies with similar *Q* values were selected, and their mean values and standard deviations were calculated from 2 to 7 values depending on the frequency. As shown in Table 1, when the standard deviation (column 2) was less than 0.5%, the mean value (column 1) was retained as the resonant frequencies extracted from the experiment.

**Figure 2:**
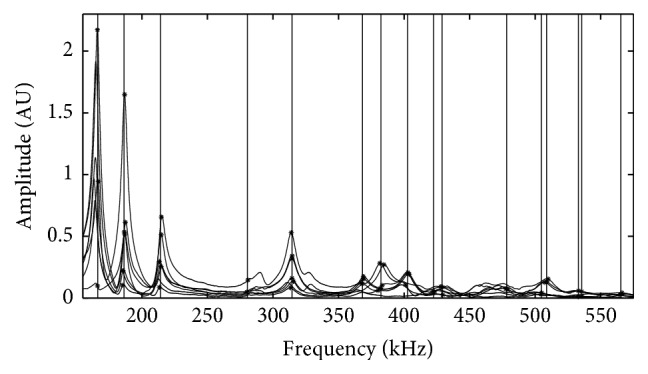
Measured frequency response of the dentin specimen between 155 and 575 kHz and the 16 resonant frequencies distributions. *∗*: the calculated resonant frequencies presented at least two times. Vertical line: the mean values of the resonant frequencies.

Figure 2 shows the measured frequency responses of the dentin specimen between 155 and 575 kHz. The 16 calculated resonant frequencies, presented at least two times, are represented as *∗*. The mean values are represented as the vertical line.

### 3.2. Results of the Inverse Problem

After nonlinear optimization, the theoretical values of resonant frequency were obtained and shown in column 4 of Table 2. Among the first 30 orders of the calculated resonant frequencies, 13 can be paired with the measured frequencies. The root-mean-square error between calculated and experimental frequencies was below 0.65%.

The correspondence elastic tensor is shown in matrix (20), with the unit of GPa.

(20) $$\begin{array}{c} \left(\begin{array}{cccccc} 35.27 & 14.06 & 18.63 & 0 & 0 & 0\\ 14.06 & 35.27 & 18.63 & 0 & 0 & 0\\ 18.63 & 18.63 & 27.71 & 0 & 0 & 0\\ 0 & 0 & 0 & 7.74 & 0 & 0\\ 0 & 0 & 0 & 0 & 7.74 & 0\\ 0 & 0 & 0 & 0 & 0 & 10.61 \end{array}\right). \end{array}$$

### 3.3. Results of Engineering Moduli Calculations

In accordance with (19), engineering moduli could be calculated as shown in Table 3. Column 3 also listed the engineering moduli results of Kinney et al.'s work [22].

### 3.4. Discussion

In this paper, the method of human dentin elasticity extraction based on RUS was studied. Firstly, the resonant spectroscopy between 155 and 575 kHz of a dentin specimen was obtained. The resonant frequencies were extracted by linear predictive filter and then by nonlinear least squares method (Levenberg-Marquardt method). Combined with the theoretical resonant frequencies calculated by Lagrangian variational method, inverse problem approach was introduced to obtain the complete second-order elastic tensor of the dentin sample. Young's moduli, shear moduli, and Poisson's ratios were finally calculated.

In theory, the lower order resonant modes are mostly shear modes, so, in the experiment, the shear wave ultrasound transducer could get stronger signal and more accurate frequency response [30]. As shown in Figure 2, the first three orders of resonant frequencies are more obvious and the resonance peaks are sharper, but the subsequent resonance peaks become broad due to the low quality factor of dentin. When the experimental resonant frequency was extracted, the linearly predictive filtering and nonlinear least squares optimization of the experimental frequency response could make it possible for the originally gentle and overlapping resonance peaks to be distinguished.

In general, when choosing the resonant order to be calculated, the number should be at least five times the number of unknown independent elastic constants [12, 14]. For transversely isotropic solids with five independent constants, it is reasonable to choose the theoretical value of the first 30-order resonant frequency in the calculations. During the Levenberg-Marquardt iteration, the elastic tensor changes constantly, leading to the the first 30-order calculated frequency band being smaller, in which only 13 orders could match with the first 13 out of the whole 16 measured resonant frequencies. In this paper, we compared each experimental resonance frequency with the theoretical resonance frequencies and tried to figure out all the pairings. But interestingly, in most cases, the cost function will not converge to the minimum value if the pairing is incorrect. Using this method, the error of each matched pairing between experimental resonance frequency and calculated resonance frequency was all less than 0.65%, which is in accordance with the 0.8% criterion described by Migliori and Maynard [30].

Comparing the result of this study, matrix (20), with the result of Kinney et al.'s group [22], shown as matrix (9), the original elastic tensor, there are differences between each independent elastic constant. We guessed that one reason is that the experimental resonant frequency extractions after the signal processing might be more accurate, which lead to the difference compared to the extraction without signal processing method of Kinney et al.'s work. Another reason might be the individual differences among different teeth. The differences in elastic tensors also resulted in some differences in engineering moduli, mainly on *E*_33_ and *ν*_12_.

Bernard et al. added probabilistic methods in the pairing process [13, 31], in which simulated annealing algorithm was introduced to compute the possibility of the pairing and then the calculated value could be automatically matched to the experimental value according to the probability. In our future work, we will try to introduce this algorithm into pairing process and explore the differences and advantages of present pairing methods. Moreover, we will also try to use more specimens to learn structure-function relationships and find other verification methods to RUS.

## 4. Conclusion

In conclusion, the elastic tensor, even Young's moduli, shear moduli, and Poisson's ratios of dentin specimen, can be accurately extracted by the signal processing method and inverse problem approach, which demonstrates that RUS is suitable for the mechanical properties measurement of low quality factor biomaterials and can provide a theoretical basis for the development of clinical dental restorative materials and the design of dental prostheses.

## Figures and Tables

**Table 1:** Results of 16 experimental resonant frequencies' extraction and *Q* factors.

*f* ^exp^/kHz	SD%	*Q*
166.34	0.22	45.56
186.44	0.43	46.05
214.20	0.36	45.40
280.50	0.20	47.98
314.47	0.22	42.05
368.28	0.23	46.41
382.30	0.45	44.57
402.73	0.29	48.32
422.47	0.17	46.21
428.92	0.19	40.78
478.22	0.18	44.81
504.72	0.06	42.28
508.66	0.25	45.63
532.94	0.01	53.49
535.48	0.02	39.95
565.41	0.22	48.76

**Table 2:** Results of the original calculated, experimental, and optimized calculated resonant frequencies.

Mode	Original *f*^cal^/kHz	*f* ^exp^/kHz	Final *f*^cal^/kHz	Err%
1	161.13	166.34	165.53	−0.48
2	195.31	186.44	186.62	0.10
3	220.66	214.20	213.07	−0.53
4	292.13	280.50	281.66	0.42
5	294.42	—	312.63	—
6	313.36	314.47	313.06	−0.45
7	333.39	—	315.26	—
8	366.55	—	342.12	—
9	385.12	—	350.58	—
10	412.00	368.28	368.17	−0.03
11	414.05	—	370.08	—
12	416.61	382.30	384.59	0.60
13	418.61	—	386.37	—
14	438.89	402.73	401.68	0.08
15	445.36	—	403.31	—
16	457.02	422.47	422.80	0.08
17	457.20	428.92	428.06	−0.20
18	484.41	—	430.71	—
19	484.84	—	451.03	—
20	496.81	—	453.71	—
21	498.90	—	453.81	—
22	516.59	—	476.06	—
23	528.65	478.22	477.13	−0.23
24	551.61	—	485.94	—
25	557.68	—	490.54	—
26	560.68	—	495.24	—
27	563.08	504.72	506.36	0.33
28	565.01	508.66	511.92	0.64
29	568.89	—	521.58	—
30	569.86	—	531.23	—

**Table 3:** Results of engineering moduli calculations.

	Modulus	Kinney et al. [22]
*E*_11_/GPa	22.641	25
*E*_33_/GPa	13.637	23.2
*G*_12_/GPa	10.608	8.6
*G*_23_/GPa	7.742	9.4
*ν*_12_	0.067	0.45
*ν*_31_	0.378	0.29
